# Supplementary material for: COZOID: contact zone identifier for visual analysis of protein-protein interactions
Source: BMC Bioinformatics. 2018 Apr 6;19:125. doi: 10.1186/s12859-018-2113-6 (PMC5889581; doi:10.1186/s12859-018-2113-6)
Supplement: Supplementary file 2 — Software build. Executable binary file of the software tool. (ZIP 118,784 kb) [file 12859_2018_2113_MOESM2_ESM.zip › cozoid/java/docs/beansbinding-1.2.1-doc.zip!/org/jdesktop/el/Expression.Result.html]

Expression.Result (Beans Binding)


---


|  |  |  |
| --- | --- | --- |
| **Overview** \| **Package** \| **Class** \| **Use** \| **Tree** \| **Deprecated** \| **Index** \| **Help** | |  |
| **PREV CLASS**   **NEXT CLASS** | **FRAMES**    **NO FRAMES**     **All Classes** |  |
| SUMMARY: NESTED \| FIELD \| CONSTR \| METHOD | DETAIL: FIELD \| CONSTR \| METHOD |  |


---


## org.jdesktop.el Class Expression.Result

```
java.lang.Object
  org.jdesktop.el.Expression.Result
```

**Enclosing class:**: Expression

---

``` public static final class Expression.Result extends java.lang.Object ```

---

| **Nested Class Summary** | |
| --- | --- |
| `static class` | `Expression.Result.Type` |


**Constructor Summary**

`Expression.Result(Expression.Result.Type type,
java.lang.Object result,
java.util.List<Expression.ResolvedProperty> resolvedProperties)`


| **Method Summary** | |
| --- | --- |
| `java.util.List<Expression.ResolvedProperty>` | `getResolvedProperties()` |
| `java.lang.Object` | `getResult()` |
| `Expression.Result.Type` | `getType()` |

**Methods inherited from class java.lang.Object**

`clone, equals, finalize, getClass, hashCode, notify, notifyAll, toString, wait, wait, wait`

**Constructor Detail**

### Expression.Result

```
public Expression.Result(Expression.Result.Type type,
                         java.lang.Object result,
                         java.util.List<Expression.ResolvedProperty> resolvedProperties)
```


**Method Detail**

### getType

```
public Expression.Result.Type getType()
```

---


### getResult

```
public java.lang.Object getResult()
```

---


### getResolvedProperties

```
public java.util.List<Expression.ResolvedProperty> getResolvedProperties()
```


---


|  |  |  |
| --- | --- | --- |
| **Overview** \| **Package** \| **Class** \| **Use** \| **Tree** \| **Deprecated** \| **Index** \| **Help** | |  |
| **PREV CLASS**   **NEXT CLASS** | **FRAMES**    **NO FRAMES**     **All Classes** |  |
| SUMMARY: NESTED \| FIELD \| CONSTR \| METHOD | DETAIL: FIELD \| CONSTR \| METHOD |  |


---
